# Effect of Intravenous Ketamine Infusion on Hemodynamics of Patients Undergoing Cesarean Delivery after Spinal Anaesthesia: A Randomized, Double-Blind, Controlled Trial

**DOI:** 10.4274/TJAR.2023.231231

**Published:** 2023-10-24

**Authors:** Mohamed Abdelgawad Abdelhalim Aboelsuod, Ahmed Mossad Ahmed Elnaggar, Tarek Abu Alkasem Abu Alwafa, Mostafa Mohamed Hussien Ahmed, Ahmed Salah Ahmed Elbeltagy, Mohamed Ibrahim Abdelkader Elbarbary

**Affiliations:** 1 Department of Anaesthesia, Intensive Care and Pain Management, Faculty of Medicine Al-Azhar University, Cairo, Egypt; 2 Department of Obstetric and Gynecology, Faculty of Medicine Al-Azhar University, Cairo, Egypt

**Keywords:** Hypotension, obstetric anaesthesia, pain, perioperative care, regional anaesthesia

## Abstract

**Objective:**

Hypotension is the most frequent side effect of intrathecal anaesthesia, with an incidence of more than 80%. Following neuraxial anaesthesia, perioperative shivering is a serious complication affecting 40-60% of patients undergoing surgery. This study aimed to determine the effectiveness of low-dose ketamine on blood pressure in patients undergoing cesarean delivery after spinal anaesthesia.

**Methods:**

We included 126 female patients undergoing cesarean deliveries, American Society of Anesthesiologists (ASA)-(II and III), and aged 21-40 selected from the outpatient clinics of the anaesthesia department. Patients were randomized to two groups; Group K (63 patients), who received 0.3 mg kg^-1^ of ketamine IV diluted to 10 mL, followed by an infusion of 0.1 mg kg^-1^ h^-1^. Group C (Controlled) (63 patients) received 10 mL of normal saline, followed by an infusion of 0.1 mL kg^-1^ h^-1^, which started before spinal anaesthesia.

**Results:**

Compared with the saline group, the average heart rate, blood pressure, and level of sedation were significantly higher in the ketamine group (*P* < 0.05). The ketamine group reported a significantly lower incidence of shivering (*P* < 0.01). The ketamine groups exhibited significantly less mild or severe hypotension (*P* < 0.05). There was no significant difference between the two groups in terms of nystagmus, diplopia, hallucinations, or neonatal outcomes (*P* > 0.05).

**Conclusion:**

Ketamine decreases the incidence of hypotension and shivering in patients undergoing spinal anaesthesia during cesarean delivery. In addition, it resulted in improved sedation for the mother and prolonged postoperative analgesia without neonatal illness.

**Main Points**

• The effect of ketamine on intraoperative blood pressure during cesarean delivery after spinal anaesthesia.

• Neonatal outcome after ketamine use in patients undergoing cesarean delivery after spinal anaesthesia.

• The effect of ketamine on shivering, sedation, and postoperative analgesia.

## Introduction

Hypotension is the most prevalent side effect of intrathecal anaesthesia, with an incidence of more than 80%. The negative effects of hypotension during spinal anaesthesia for cesarean delivery include reduced uteroplacental blood flow, impaired fetal oxygenation with asphyxia stress, and fetal acidosis, as well as maternal symptoms of low cardiac output, such as nausea, vomiting, dizziness, and decreased consciousness. These adverse effects can harm both the mother and newborn. Methods for preventing and managing hypotension in obstetric anaesthesia have garnered significant interest in the literature. However, there is controversy over the utility of IV fluid preload. Uterine displacement is common. Despite these precautions, it is frequently necessary to administer a vasopressor. Ephedrine effectively demonstrated effectiveness in restoring maternal arterial pressure following hypotension and is typically prescribed in such cases.^1^

Ketamine increases the release and inhibits the reuptake of catecholamines, thereby preserving arterial blood pressure and vascular resistance, making it the optimal anaesthetic agent for hypotensive conditions.^2^ The sympathetic nervous system is stimulated by ketamine, which results in an elevated heart rate (HR) and hypertension. It can raise intraocular and intracranial pressures, and its use is restricted in conditions where such an increase in pressure could be harmful (eye injury, head trauma, vascular disease, and hydrocephalus, for example).^3^

Shivering during surgery is a prevalent issue in anaesthesia practice, resulting in discomfort and life-threatening issues if not effectively controlled and prevented, especially in cardiorespiratory patients. Surgical patients may experience shivering for various reasons, including surgery, anaesthesia, skin exposure in a cool operating room, and receiving unwarmed fluids. Numerous pharmacological and non-pharmacological methods exist to prevent and treat this issue. Methods for preventing and treating shivering include prewarming the patient for 15 min prior to anaesthetic administration and administering modest doses (e.g., ketamine, clonidine, pethidine, dexamethasone, dexmedetomidine, tramadol, and magnesium sulfate).^2^ Therefore, beneficial analgesic effects can be achieved without psychoactive side effects such as hallucinations and blockade of excitatory synaptic activity caused by loss of responsiveness associated with clinical ketamine anaesthesia.^4^ However, subsequent research revealed that ketamine exhibits several different molecular effects and plays a role in the management of a wide range of conditions, including acute and chronic pain, and rapidly acting antidepressant.^5^

We hypothesize that ketamine decreases the incidence of spinal-induced hypotension in cesarean delivery by a ketamine sympathhomimetic effect.

This study aimed to determine the effectiveness of ketamine infusion on hemodynamic parameters in patients undergoing cesarean delivery after spinal anaesthesia.

## Methods

We included 126 female patients, aged 21-41 years, undergoing cesarean deliveries with an American Society of Anesthesiologists (ASA)-(II and III). Subjects were recruited from the outpatient clinics of the Anaesthesia Department Outpatient Clinics in Al-Azhar University Hospitals from September 2022 to February 2023. Patients were randomly assigned to two groups; Group K (63 patients), who received 0.3 mg kg^-1^ of ketamine IV diluted to 10 mL, followed by an infusion of 0.1 mg kg^-1^ h^-1^ as 20 mL solution. Group C (Controlled) (63 patients) received 10 mL of normal saline, followed by an infusion of 0.1 mL kg^-1^ h^-1^ as a 20 mL solution.

The type of study: Randomized, double-blind, prospective, controlled study.

### Study Outcomes

Primary outcomes: Hemodynamic parameters (MAP and HR).

### Secondary outcomes

1. Incidence of intraoperative shivering

2. Postoperative pain was assessed by VAS score.

3. Sedation score between groups.

4. Fetuse evaluated using the Apgar score.

5. Postoperative side effects include nausea, vomiting, nystagmus, diplopia, and hallucinations.

### Ethical Considerations

The Research Ethics Committee approved the study protocol at Al-Azhar University (approval no: 00328/2022). Written informed consent was obtained from each patient before the operation. This research is registered in the Clinical Trials Register (NCT05865080).

### Inclusion criteria

Female patients and full-term, between (21 and 40), with (ASA)-II or III, and undergoing a cesarean section.

### Exclusion criteria

1. Twins and preterm birth.

2. Hypertensive and preeclamptic patients.

3. Morbidly obese patients.

4. Spinal anaesthesia contraindication because the patient refused severe mitral or tricuspid stenosis and local sepsis.

### Randomization

**Figure 1:**
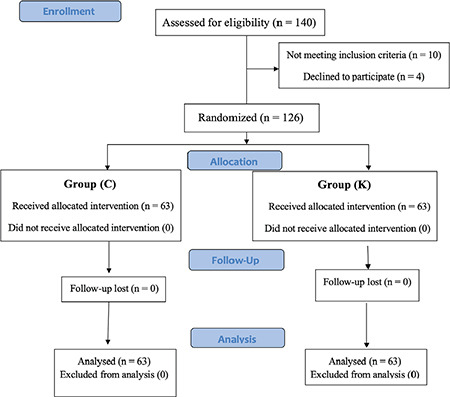
CONSORT diagram chart

Ten minutes before the start of anaesthesia, the patients were equally randomized into two groups using computer-generated random numbers placed in separate opaque envelopes. The researcher opened the envelopes immediately before administering spinal anaesthesia, as depicted in the consort chart (Figure 1). An anaesthetist blinded to the study groups prepared two syringes, one containing ketamine (5 mg mL^-1^, Ketalar, Pfizer, New York) and the other containing 0.9% saline. Both syringes were labeled “study drug” to maintain the double-blind design of the study.

### Anaesthetic procedure:

All patients underwent preoperative planning before surgery, which included history taking, tests, and examinations. The patient was connected to standard monitoring devices such as noninvasive arterial blood pressure, electrocardiogram, and pulse oximeter, with baseline parameters measured and recorded in the pre-operative holding area. A wide-pore IV cannula was placed with preoperative Ringer lactate (500 mL) as preload. No pre-medical treatment was administered. At the L4-5 level, spinal anaesthesia was administered using a paramedian approach while seated. A 25 G Quinke needle and 2 mL of 0.5% heavy bupivacaine mixed with 25 g of fentanyl were used. A 2-liter nasal cannula was used to connect all patients. Ketamine was administered prior to the administration of spinal anaesthesia and was discontinued at the end of surgery.

### Surgical procedures

Before administering spinal anaesthesia, the obstetrician and nurse disinfected their hands with betadine and sterilized the patient. The assessment of the patient’s lower limb motor block and bilateral loss of sensation with a pinprick to the T4 dermatomes indicated that the patient had adequate surgical anaesthesia. After ensuring the absence of sensation, the operation began, and the newborn was evaluated at 1 and 5 min using the Apgar score by the pediatrician. Following delivery and clamping of the umbilical cord, oxytocin was administered. According to the obstetrician’s recommendation, incremental doses of 10 units of oxytocin were administered, followed by increments of 2 units, depending on the contractility of the uterus. After completion of the operation, the patient was transferred to the recovery room.

### Measurements

1. The baseline data included the duration of the procedure, the patient’s height, weight, age, gestational age, and an indication of the cesarean section.

2. Intraoperative hemodynamics.

3. Incidence of shivering among groups.

4. Evaluation of sedation by Ramsay sedation score at 5, 10, 20, 30, and 40 min after surgery.

5. At four, eight, twelve, sixteen, twenty, and twenty-four hours, the visual analog scale (VAS) was evaluated.

6. Fetus Apgar score in the 1^st^ and 5^th^ min.

7. Postoperative side effects, such as nausea, vomiting, nystagmus, diplopia, and hallucinations.

### Ramsay sedation score

1. Anxious, agitated, and restless.

2. Oriented, tranquil.

3. Responds to commands.

4. Brisk response to light glabellar tap.

5. Sluggish response to light glabellar tap.

6. No response (deep sedation).

### Sample size justification

Using Epi-info TM version 7.2.4.0 (2020), the sample size was determined on the basis of the following factors:

• Level of tow-side confidence: 95%

• 80% of the test power.

• 5% error rate.

According to the findings of the study by Salah and Alansary^6^ on hemodynamic affection, a minimum sample size of 140 subjects was required, plus an additional 15% (or approximately 24 patients) to account for dropouts. Therefore, the study included 63 patients in each group to test the hypothesis.

### Statistical Analysis

The collected data were coded, processed, and analyzed using SPSS (Version 25) for Windows. Descriptive statistics were calculated to include mean, standard deviation, median, range, and percentage. For continuous variables, independent t-tests were performed to compare the means of normally distributed data. The Mann-Whitney U test was used to compare the median differences in non-normally distributed data, whereas the chi-square test was used for categorical data. The t-test and Wilcoxon signed-rank test were used for independent groups. The level of statistical significance was set at* P* values <0.05.

## Results

One hundred forty patients passed the eligibility criteria. There were 14 patients excluded from the exclusion criteria. A total of 126 patients were randomly assigned to two groups, as depicted in the CONSORT flowchart (Figure 1).

There were statistically significant differences between the groups with regard to age, weight, ASA, gestational age, height, length of the procedure, and an indication of cesarean section (*P* > 0.05) as shown in Table 1.

According to Tables 2 and 3, there were statistically significant differences between the groups in terms of HR and blood pressure (*P* < 0.05), with the ketamine groups exhibiting greater hemodynamic stability.

There were statistically significant differences between the groups regarding intraoperative sedation and the frequency of shivering (*P* < 0.05), as depicted in Table 4. There were statistically significant differences between the groups in terms of pain score (VAS) postoperatively, which was lower in the ketamine groups (*P* < 0.05), as illustrated in (Table 5).

There were statistically significant differences between the groups in terms of hypotension, nausea, and vomiting (Figure 1).

**Figure 2:**
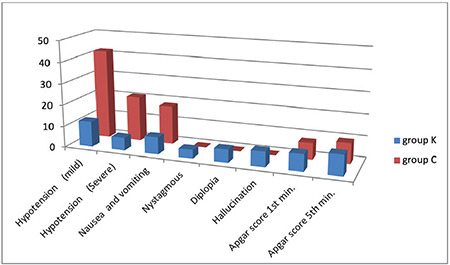
The two groups’ side effects.

There was no significant difference between the groups in terms of nystagmus, diplopia, hallucinations, or neonatal outcomes (*P* > 0.05) with respect to postoperative side effects, as demonstrated in (Figure 2).

## Discussion

Spinal anaesthesia applications, particularly during cesarean section in pregnant women, have been found to cause both hypotension and tremor with sympathetic blockade and are accompanied by some complications. This study avoided the common adverse effects associated with obstetric anaesthesia.

When compared with the control group, the ketamine patients in the current study demonstrated hemodynamic stability in terms of mean blood pressure and HR (*P* < 0.05). These findings agree with those of Salah and Alansary^6^ study, which reported that a minimal amount of ketamine could be used to prevent hypotension following intrathecal anaesthesia in CS.

With a frequency of between 40% and 60% in patients undergoing surgery, perioperative shivering is a severe adverse effect frequently following neuraxial anaesthesia. In particular, in patients with cardiorespiratory issues, it causes excruciating discomfort and negative outcomes. Many techniques have been devised to avoid and manage shivering during and after neuraxial anaesthesia.^7,8^

In spinal anaesthesia, hypotension is caused by sympathetic nervous system blockade, and aortic and inferior vena cava compression during pregnancy is typically treated with ephedrine, intravenous fluid, and left lateral tilt.^9^

In the present study, 38.1% of patients in the saline group experienced postoperative shivering, compared with 7.94% in the ketamine group. The percentage of patients recovering from anaesthesia who experienced postoperative shivering ranged from 5-65%,^10^ and numerous studies have reported that ketamine plays a role in reducing postsurgical shivering.^11^

Thangavelu et al.,^12^ reported that only 4 cases (13.79%) of intraoperative shivering were observed in the ketamine group compared with 80 cases (58.06%) in the group receiving saline. In addition, the ketamine group showed significantly less postoperative shivering than the saline group. A small bolus of low-dose ketamine followed by an infusion prevented intraoperative and postoperative shivering. In the current study, intraoperative sedation was superior in the ketamine group compared with that in the control group. Also postoperative analgesia was better in the ketamine group than in the control group. Brinck et al.^13^ studies on intravenous ketamine during surgery to treat severe postoperative pain in adults revealed a reduction in opioid dependance (an average decrease of 14.38 mg of intravenous morphine equivalents in 24 h).

Pendi et al.,^14^ studies on patients who underwent spine surgery using perioperative ketamine as an analgesic and found that it reduced opioid-related side effects such as postoperative nausea and vomiting and respiratory sedation and improved engagement in recovery-oriented activities such as postoperative physiotherapy. In this study, there were significant differences in VAS between the groups postoperatively, with the ketamine group experiencing prolonged analgesia. These findings are consistent with those of Seman et al.,^15^ studies in patients with morbid obesity undergoing laparoscopic gastric bypass surgery. They reported that infusions of ketamine significantly reduced the need for opioids in the ketamine group compared with the control group.

In the current study, 8 (14.28%) patients in the ketamine group and 18 (60.32%) patients in the saline group experienced nausea and vomiting. Nystagmus was reported in four patients (6.3%), diplopia in six (9.5%) patients and hallucinations in seven patients (11.11%) in the ketamine group, but none of these side effects were reported in the saline group. In more than 80% of cases, the most frequent side effect of intrathecal anaesthesia is hypotension, which can negatively impact uterine blood flow and the health and outcome of the fetus as measured by Apgar scores.^16^ In the compared groups of this study, Apgar scores remained unchanged.

Adhikari et al.,^17^ illustrated that in patients who undergo nonelective cesarean deliveries, intravenous administration of a small dose of ketamine before surgical incision significantly decreases the need for opioid usage in the first 24 h post-surgery.

Karacaer et al.,^18^ reported that a continuous infusion of ketamine and deflurane inhalation in patients with chronic obstructive pulmonary disease during one-lung ventilation increased arterial oxygenation and decreased shunting by ketamine effect on the catecholamine reuptake inhibitor mechanism. Therefore, there was no risk of respiratory depression when using a small dose of ketamine, and there were no cases of desaturation.

In this study, the incidence of mild or severe hypotension was significantly lower in the ketamine group than in the saline group.

Dhiman et al.,^19^ reported that a small nebulized dose of ketamine with dexmedetomidine was superior to sedation with enhanced ease of intravenous line and postoperative analgesia in children.

Ketamine increases the release and inhibits the reuptake of catecholamines in circulation, thereby aiding in the preservation of vascular resistance and arterial blood pressure, making it the optimal anaesthetic method for hypotensive patients.^20^

Numerous clinical and pharmacological properties of ketamine have recently been reported. There are numerous applications of ketamine in anaesthesia, pain management, and intensive care.^21^

Intravenous low-dose ketamine combined with midazolam for sedation during spinal anaesthesia for elective cesarean section provides more effective and long-lasting pain relief than the control group.^22^

Spinal anaesthesia applications, especially during cesarean section in pregnant women, cause both hypotension and tremor with sympathetic blockade and result in many complications. This study will provide an opportunity to avoid undesirable effects frequently encountered in obstetric anaesthesia.

### Study Limitations

In this research, the study was conducted on some cases, and patient satisfaction was not evaluated.

## Conclusion

Ketamine decreases the incidence of hypotension and shivering in patients undergoing spinal anaesthesia during cesarean delivery. In addition, it resulted in improved sedation for the mother and prolonged postoperative analgesia without neonatal illness.

## Figures and Tables

**Table 1:**
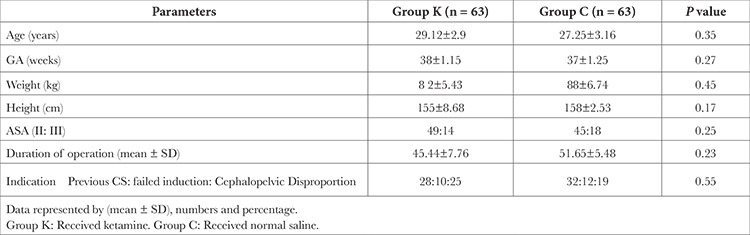
Basic Data on the Study Population

**Table 2:**
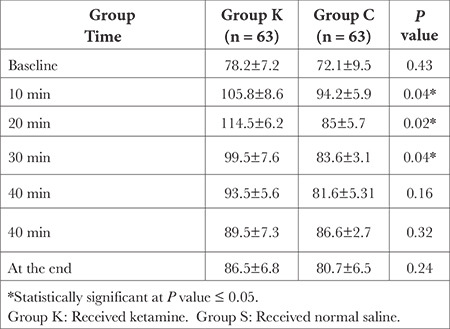
Heart Rate Changes at Different Time (Mean ± SD) in (b min^-1^)

**Table 3:**
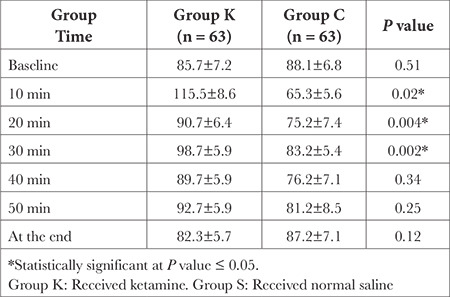
Mean Arterial Blood Pressure Changes (Mean ± SD) in mmHg

**Table 4:**
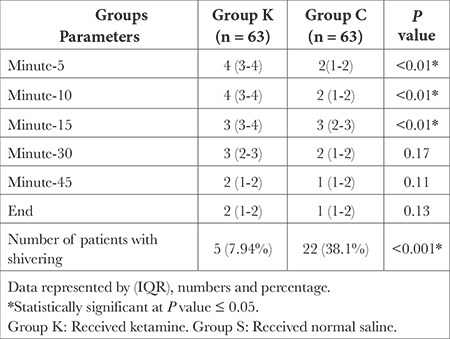
Sedation Scores and Number of Patients with Shivering

**Table 5:**
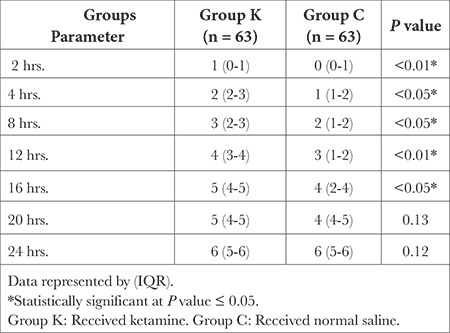
Postoperative Pain Score (VAS) Between Two Groups
